# Rhizoma drynariae total flavonoids combined with calcium carbonate ameliorates bone loss in experimentally induced Osteoporosis in rats via the regulation of Wnt3a/β-catenin pathway

**DOI:** 10.1186/s13018-021-02842-3

**Published:** 2021-12-04

**Authors:** Yimei Hu, Panyun Mu, Xu Ma, Jingru Shi, Zhendong Zhong, Lingyuan Huang

**Affiliations:** 1 Affiliated Hospital of Chengdu University of Traditional Chinese Medicine, Chengdu, 610075 Sichuan China; 2 Chengdu University of Traditional Chinese Medicine, Chengdu, 610075 Sichuan China; 3 Institute for Laboratory Animal Research, Sichuan Academy of Medical Sciences & Sichuan Provincial People’s Hospital, Chengdu, China; 4 Chengdu Lilai Biotechnology Co., LTD, Chengdu, China

**Keywords:** Osteoporosis, *Rhizoma drynariae* total flavonoids, Calcium carbonate, Wnt3a/β-catenin

## Abstract

**Background:**

*Rhizoma drynariae*, a traditional Chinese herb, is commonly used in treatment of bone healing in osteoporotic fractures. However, whether the *Rhizoma drynariae* total flavonoids (RDTF) can promote the absorption of calcium and enhance the bone formation is unclear. The aim of the present study was to investigate the preventive effects of RDTF combined with calcium carbonate (CaCO_3_) on estrogen deficiency-induced bone loss.

**Methods:**

Three-month-old Sprague–Dawley rats were ovariectomized (OVX) and then treated with CaCO_3,_ RDTF, and their admixtures for ten weeks, respectively. The bone trabecular microstructure, bone histopathological examination, and serum biomarkers of bone formation and resorption were determined in the rat femur tissue. The contents of osteoprotegerin (OPG), receptor activator of the NF-κB (RANK), and its ligand (RANKL) in marrow were analyzed by ELISA, and the protein expressions of Wnt3a, β-catenin, and phosphorylated β-catenin (p-β-catenin) were analyzed by Western blot. Statistical analysis was conducted by using one-way analysis of variance (ANOVA) followed by LSD post hoc analysis or independent samples t test using the scientific statistic software SPSS version 20.0

**Results:**

RDTF combined with CaCO_3_ could promote osteosis and ameliorate bone loss to improve the repair of cracked bone trabeculae of OVX rats. Furthermore, RDTF combined with CaCO_3_ also could prevent OVX-induced decrease in collagen fibers in the femoral tissue of ovariectomized rats and promote the regeneration of new bone or cartilage tissue, while CaCO_3_ supplementation promoted the increase in bone mineral content. Nevertheless, there was no difference in the expression of Wnt3a, β-catenin and p-β-catenin between osteopenic rats and RDTF treated rats, but RDTF combined with CaCO_3_ could activate the Wnt3a/β-catenin pathway.

**Conclusions:**

RDTF combined with CaCO_3_ could ameliorate estrogen deficiency-induced bone loss via the regulation of Wnt3a/β-catenin pathway.

## Introduction

Osteoporosis (OP) is a systemic skeletal disease characterized by low bone mass and micro-architectural deterioration of the trabecular and envelop of cortical bone, which increase bone fragility and susceptibility [1]. Estrogen deficiency in postmenopausal women results in excessive bone resorption without adequate new bone formation, followed by bone loss and osteoporosis [2]. Menopausal hormone therapy, as the major therapy for the prevention and treatment of postmenopausal osteoporosis [3, 4], has been introduced in clinical treatments of OP, but it only alleviates a small part of the symptoms of OP and related fractures, even it was associated with an increased risk of breast cancer, ovarian cancer, and endometrial cancer development in postmenopausal women [5–7].

Calcium is an essential element in bone formation and the key component of hydroxyapatite, which can play a single therapeutic role in osteoporosis [8, 9]. Also, recent studies on no hormone replacement therapy have shown that calcium is the simplest and cheapest strategies to treat and prevent osteoporosis [10]. Among the women with low risk of fracture, optimizing calcium intake is the best way to prevent osteoporotic fracture in postmenopausal women [11]. Therefore, calcium supplementation may be an effective way to treat OP. However, calcium supplementation alone has no effect on the prevention of OP and even aggravates the risk of fracture ^8^.

In recent years, dietary phytoestrogens have been proved to be identified as a safe and effective candidate drug with estrogenic properties in the treatment of postmenopausal osteoporosis [12]. Previous studies have shown that phytoestrogens, such as soy isoflavones [13, 14], lignans [15], and flavonoids [16, 17], can reduce bone loss associated with estrogen deficiency in both animal and human studies. *Rhizoma drynariae* is a kind of traditional Chinese medicine commonly used in orthopedics [18]. Its main bioactive constituents of is flavonoids, which is commonly used in the treatment of fractures. Because of its potential roles in preventing osteoporosis and promoting the differentiation of osteoblasts [19], it has attracted much attention. Thereinto, naringin and neoeriocitrin are the two major flavonoid compounds with the highest concentrations in *Rhizoma drynariae* [20].

Although previous studies have confirmed the efficacy of *rhizoma drynariae* total flavonoids (RDTF) in treating OP [21, 22], its mechanism is still difficult to be fully elucidated due to its complex components and numerous targets. Recent studies have corroborated that *rhizoma drynariae* could improve the level of calcium and phosphorus in blood, activate the osteoblast, and increase the bone mineral density of thighbone [16, 17]. The previous study showed that RDTF combined with calcium could improve OP through relieving oxidative stress [23]. However, it is not clear whether the *rhizoma drynariae* total flavonoids (RDTF) combined with calcium can promote the bone formation. Therefore, it is necessary to study the effects of RDTF combined with calcium on bone loss in osteopenic rats and reveal the molecular mechanisms of RDTF.

## Material and methods

### RDTF and calcium carbonate

RDTF were purchased from Beijing Qihuang Pharmaceutical Manufacturing Co., Ltd. (National Medicine Permit No. Z20030007, number of production: 04,080,081, the content of RDTF ≥ 80%). Calcium supplement: it is recommended that women take 1500 mg of CaCO_3_ [10]. The recommended dose calculated in rats was 20 mg daily given as CaCO_3_. 20 g CaCO_3_ was dissolved in 1000 ml water, and each rat received 1 ml suspension every day by oral.

### Animals and treatments

Three-month-old Sprague–Dawley specific pathogen-free female rats (250 ± 20 g) purchased from Chengdu DOSSY Experimental Animals Co., Ltd (Chengdu, China) were subjected to adaptive feeding for 7 days under standard housing conditions prior to the animal experiments. The acclimatized rats underwent either bilateral laparotomy (Sham, n = 6) or bilateral ovariectomy (OVX, n = 24), according to the method described by Elkomy [24]. Four week after recovering from surgery, the ovariectomized rats were randomly divided into five groups: OVX with vehicle (OVX, n = 6); OVX with CaCO_3_ (n = 6, 20 mg/kg/day); OVX with RDTF (n = 6, 50 mg/kg/day); OVX with RDTF and CaCO_3_ (n = 6, RDTF: 50 mg/kg/d, CaCO_3_: 20 mg/kg/d). Vehicle, CaCO_3_ and RDTF were all administered orally through a custom-made stomach tube, which lasted for 10 weeks. The bone mineral content (BMC) and bone mineral density (BMD) were measured by dual-energy X-ray absorptiometry (DEXA) two days before the animals were euthanized. After the rats were anesthetized with pentobarbital sodium, a laparotomy was performed and blood samples were collected by abdominal aorta puncture from each anesthetized rat. Serum was then prepared by centrifugation (3000 g for 10 min at 4 °C) for biochemical determinations.

All surgical interventions and postoperative animal care were approved by the ethics committee of West China Hospital, Sichuan University, with an associated permit numbers (20211152A).

### DEXA and micro‑computerized tomography analysis

Methods for measuring trabecular microstructure parameters are as previously described [25]. Brief, femurs were cleaned of soft tissue and fixed in 4% paraformaldehyde. Samples were imaged on Inveon micro-CT (Siemens, Germany) with parameters of 9-µm voxel size, 50 kVp, 200 µA, using a 0.5-mm aluminum filter. Mineral density was determined by calibration of images against 2-mm-diameter hydroxyapatite rods (0.25 and 0.75 gHA/cm^3^). A beam-hardening correction algorithm was applied prior to image reconstruction. Trabecular bone analysis was performed at the distal femoral metaphysis. The region of interest was selected 360 µm proximally to the distal growth plate and extended proximally 1.8 mm. The trabecular region was selected by contouring. An adaptive threshold (using the mean maximum and minimum pixel intensity values of the surrounding ten pixels) was used to identify trabecular bone, and an erosion of 1 pixel was performed to eliminate partial volume effects. This region of trabecular bone was used to determine bone mineral content (BMC), bone mineral density (BMD), tissue volume (TV), bone volume (BV), bone volume fraction (BV/TV), trabecular thickness (Tb.Th), and trabecular separation (Tb.Sp) with purpose-designed software (enCORE™ 2006, GE Healthcare, Madison, WI, USA).

### Histopathological examination

Femur bones were dissected and fixed in 4% paraformaldehyde after the removal of soft tissue. After decalcified in formic acid for 3 weeks, the femurs were paraffin-embedded, sectioned, and were kept for histopathological examination using hematoxylin and eosin (H&E) stain to observe the bone microstructure of the femur. Meanwhile, the femurs were also stained with Masson's trichrome staining kit (Solarbio, Beijing, China) in accordance with the manufacturer’s protocol to detect collagen fibers and new bone in the femur. The area of collagen fibers was assessed with the Image J analysis software system following Masson staining.

### Assay for serum chemistry

Serum calcium (S-Ca), serum phosphorus (S-P), and alkaline phosphatase (ALP) concentrations were measured by standard colorimetric methods using commercial kits (Nanjing Jiancheng Bioengineering Ltd., China) and analyzed with a multi-functional microplate spectrophotometer (SpectraMax M4, Molecular Devices, USA). Serum C-terminal telopeptide of type I collagen (S-CTX), osteocalcin (BGP), and tartrate-resistant acid phosphatase (TRAP) levels in serum and the receptor activator of the NF-κB ligand (RANKL), osteoprotegerin (OPG), and RANK levels in marrow were determined using rat ELISA kits (Biocalvin, Suzhou, China).

### Western blotting

Bone marrow were flush out by a small amount of PBS fluid. After bone marrow collection, the marrow homogenate of rat was prepared and then lysed with RIPA buffer in the presence of 1% protease inhibitor cocktail (Roche, Basel, Switzerland). Protein from bone marrow homogenate was also extracted using RIPA buffer. The supernatant was collected after centrifugation at 12,000 g and 4 °C for 30 min. Protein concentration was quantified with a BCA Protein Assay Kit (Generay, Shanghai, China). After denatured in boiling for 5 min in SDS sample buffer, 40 μg of total protein was separated by 6%–15% SDS-PAGE, blotted onto PVDF membranes, and then probed with the following antibodies (Cell Signaling Technology, Danvers, MA, USA): monoclonal anti-Wnt3a antibody (1:1000), monoclonal anti-β-catenin antibody (1:1000), monoclonal anti-phosphorylated β-catenin (p-β-catenin) antibody (1:1000), and mouse anti-β-actin antibody (1:1000), conjugated to horseradish peroxidase, were used as secondary antibodies (1:5000). Protein bands were visualized by incubation with BeyoECL Plus (P0018, Beyotime, China) for 1 min and imaged by a Gel Image System (Tanon, 5200, China). Densitometry was performed by using Image J software.

### Statistical analysis

All data are summarized as mean ± standard deviation (SD) with at least three independent replicates. Statistical analysis was conducted by using one-way analysis of variance (ANOVA) followed by LSD post hoc analysis or independent samples t-test using the scientific statistic software SPSS version 20.0 (SPSS Inc., Chicago, IL, USA). *P* < 0.05 was considered statistically significant.

## Results

### Effect of RDTF and CaCO_3_ on the trabecular microstructure in OVX rats

To investigate the effect of RDTF and CaCO_3_ on the trabecular bone microstructure of OVX rats, CaCO_3_ (20 mg/kg/d), RDTF (50 mg/kg/d), and their mixed preparations were administered to each OVX rat every day beginning at 5th weeks after surgery. The anesthetized rats were sacrificed after 10 weeks treatment, respectively.

DEXA scans analysis of the distal femur trabecular bone showed that the bone microstructure of the OVX group deteriorated, as evaluated by decreasing in BMD, BMC, BV/TV, Tb.Th parameters and increasing in Tb.Sp parameters compared with the sham operation group (Table 1 and Figure S1). Compared with the OVX group, RDTF, CaCO_3_, and their combined treatment notably promoted the increase in bone density (BMD) and bone volume fraction (BV/TV). There was no difference in bone mineral content (BMC) between OVX group and RDTF group, but CaCO_3_ and the combined treatment with RDTF and CaCO_3_ significantly promoted the increase of BMC. Furthermore, the effect of increasing BMC of their mixed preparations was better than RDTF (*P* < 0.05). Importantly, we found that treated with CaCO_3_ alone could not improve the bone trabecular thickness (Tb.Th) and trabecular separation (Tb.Sp) of OVX rats, but treated with RDTF alone or combination of RDTF and CaCO_3_ significantly increased Tb.Th and decreased Tb.Sp of ovariectomized rats. These results suggest that RDTF, CaCO_3_, and their mixed preparations could significantly improve the trabecular microstructure of ovariectomized rats. Moreover, the effect of combined treatment with RDTF and CaCO_3_ on bone microstructure was better than exclusive treatment with CaCO_3_ or RDTF.

**Table 1:** Effect of RDTF and CaCO_3_ on the trabecular microstructure of OVX rats

Parameters	Sham	OVX	CaCO_3_	RDTF	RDTF + CaCO_3_	
BMD (g/cm^2^)	0.258 ± 0.004	0.189 ± 0.005***	0.236 ± 0.009**^###^	0.233 ± 0.014**^###^	0.248 ± 0.003^###^	
BMC (g/cm^2^)	0.087 ± 0.001	0.070 ± 0.002***	0.074 ± 0.003***^#^	0.073 ± 0.001***^&^	0.077 ± 0.001***^###^	
BV/TV (%)	0.502 ± 0.019	0.287 ± 0.026***	0.399 ± 0.021***^###&^	0.415 ± 0.009***^###^	0.445 ± 0.007**^###^	
Tb.Th (mm)	0.122 ± 0.025	0.072 ± 0.015***	0.085 ± 0.009**	0.120 ± 0.014^#^	0.122 ± 0.010^###^	
Tb.Sp (mm)	0.141 ± 0.012	0.173 ± 0.008**	0.170 ± 0.011**^&^	0.152 ± 0.003^#^	0.145 ± 0.008^##^	

BMD: bone mineral density; BMC: bone mineral content; BV/TV: bone volume/tissue volume; Tb.Th: trabecular thickness; Tb.Sp: trabecular separation

*P < 0.05, **P < 0.01, ***P < 0.001 vs. Sham. ^#^*P* < 0.05, ^##^*P* < 0.01, ^###^*P* < 0.001 vs. OVX. ^&^*P* < 0.05 vs RDTF + CaCO_3_

### Anti-osteoporosis effects of RDTF and CaCO_3_ on ovariectomized rats

**Fig. 1:**
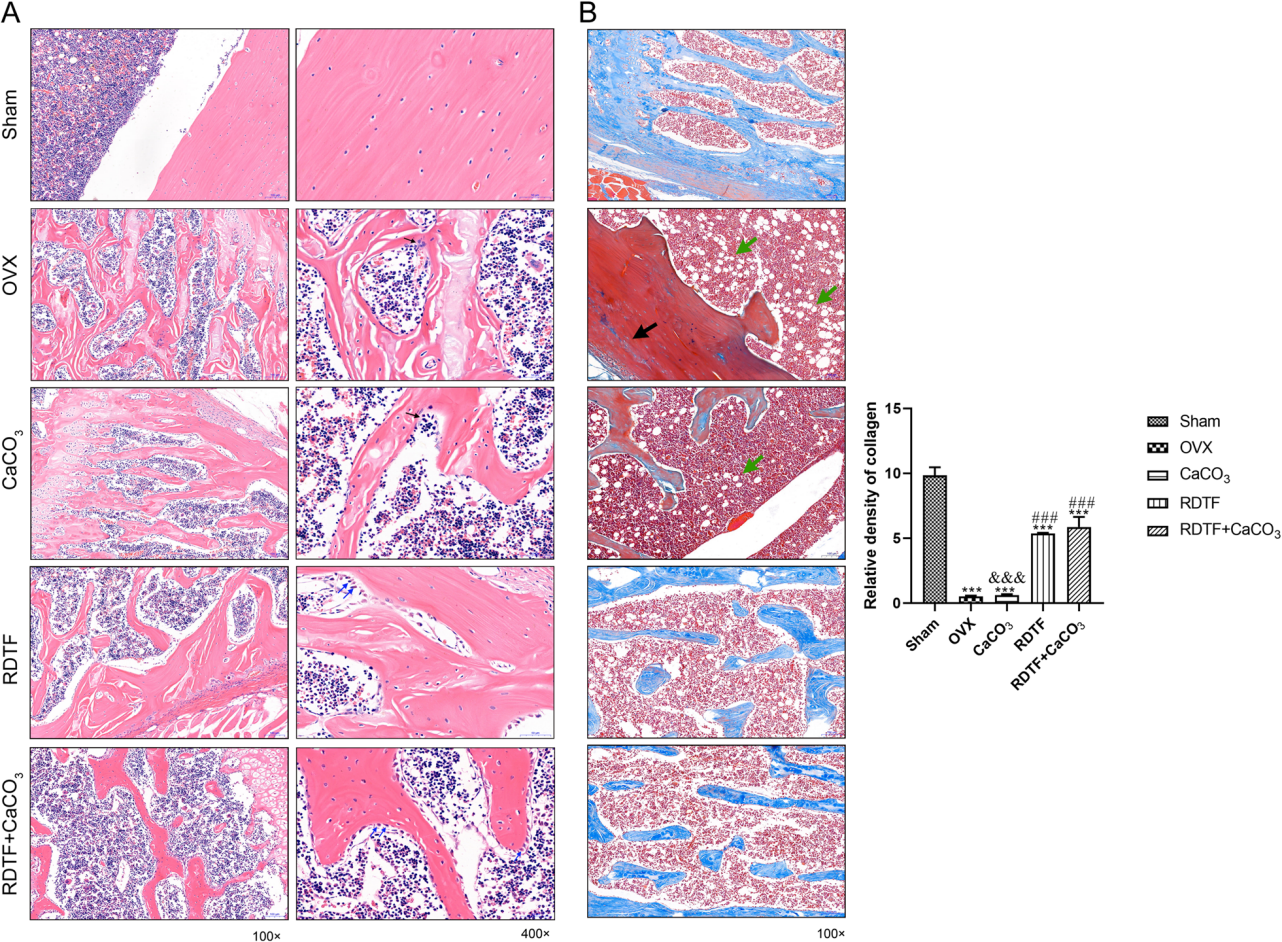
Effect of RDTF and calcium on bone histopathological examination of OVX-induced osteoporotic rats. **a** H&E staining for femur. Bone tissue of sham rats showed normal epiphyseal structure with normal bone trabeculae; OVX rats showed apparent crack and thinning of bone trabeculae with the presence of multi-nucleated osteoclasts on the surface of the trabeculae (black arrows); CaCO_3_-treated rats showed crack and thinning of bone trabeculae same as OVX rats; RDTF-treated rats showed increased thickness of the trabeculae with activation and proliferation of osteoblasts which appeared plump with abundant basophilic cytoplasm (blue arrows); RDTF combined with CaCO_3_-treated rats showed compact bone trabeculae with remarkable activation and proliferation of osteoblasts. **b** Masson’s trichrome staining. Sham rats showed abundant collagen fibers and new bone or cartilage in femoral tissue; OVX rats and CaCO_3_ treated rats both showed apparent absence of collagen fibers and cartilage in femoral tissue; RDTF and RDTF combined with CaCO3 treated rats both showed apparent increase of collagen fibers and cartilage in femoral tissue. Black arrow: less cartilage (or collagen fibers); green arrow: fat droplets in the marrow cavity. Quantification of collagen fiber density was analyzed by optical density. *P < 0.05, **P < 0.01, ***P < 0.001 vs. Sham. ^#^*P* < 0.05, ^##^*P* < 0.01, ^###^*P* < 0.001 vs. OVX. ^&^*P* < 0.05, ^&&^*P* < 0.01, ^&&&^*P* < 0.001 vs RDTF + CaCO_3_

To evaluate the effects of RDTF and CaCO_3_ on the trabecular microstructure of OVX rats, the H&E staining was investigated (Fig. 1a). Compared with the sham operation group, the trabecular structure of femur in OVX rats was sparse, fractured, spacing-enlarged, and area-diminished trabecular. After 10-week treatment of OVX rats, the CaCO_3_ treatment group exhibited the same osteoporosis as OVX group, with a sparse and fractured trabecular structure of femur. However, the RDTF treatment group exhibited partial trabecular restoration, and the combined treatment with RDTF and CaCO_3_ group showed almost complete recovery of normal structure after 10 weeks treatment. These data consisted of the DEXA scan, which indicated that RDTF and combined with CaCO_3_ promoted the repair of cracked bone trabeculae of ovariectomized rats, and RDTF combined with CaCO_3_ showed a better effect on fracture repair.

Masson’s staining indicated that the contents of collagen fibers and the levels of new bone or cartilage in femoral tissue of ovariectomized rats were decreased (Fig. 1b). There was no significant improvement in the content of collagen fibers and the content of new bone or cartilage induced by OVX after treatment with CaCO_3_. However, RDTF and RDTF combined with CaCO_3_ for 10 weeks could restore the level of collagen fibers in the femoral tissue of ovariectomized rats and promote the regeneration of new bone or cartilage tissue.

### Effect of RDTF and CaCO_3_ on bone formation and resorption biomarkers

In ovariectomized female rats, the decrease in estrogen promotes the increase in osteoblast and osteoclast activity and induces the increase in bone turnover rate, with the abnormal increase in bone formation and resorption biomarkers ALP, TRACP, BGP, and S-CTX in blood [21]. The biochemical serum parameters from all groups are shown in Table 2. The OVX treatment did not alter the serum levels of Ca, P, and BGP between all groups (*P* > 0.05). Nevertheless, the levels of ALP, TRACP, and S-CTX in the OVX group were significantly increased compared with the Sham group (*P* < 0.001). Treatment with CaCO_3_, RDTF, or RDTF combined with CaCO_3_ all significantly prevented the OVX-induced increase in ALP level (*P* < 0.001), and the effect of RDTF combined with CaCO_3_ on decreasing ALP level was significantly better than RDTF alone. Although treatment with CaCO_3_ and RDTF decreased the level of TRACP and S-CTX, but there was no significant difference in the level of TRACP and S-CTX between OVX group and either treatment with CaCO_3_ or RDTF alone (*P* > 0.05). However, RDTF combined with CaCO_3_ significantly prevented the OVX-induced increase in TRACP (*P* < 0.05) and S-CTX levels (*P* < 0.01). These results indicated that RDTF combined with CaCO_3_ could prevent the OVX-induced increase in the bone turnover rate in rats.

**Table 2:** Effect of RDTF and calcium on biochemical parameters in serum of rats

Parameters	Sham	OVX	CaCO_3_	RDTF	RDTF + CaCO_3_	
S-Ca (mmol/l)	2.53 ± 0.04	2.59 ± 0.04	2.57 ± 0.04	2.56 ± 0.07	2.53 ± 0.08	
S-P (mmol/l)	1.64 ± 0.05	1.70 ± 0.02	1.63 ± 0.09	1.69 ± 0.07	1.66 ± 0.12	
ALP (U/100 ml)	89.69 ± 3.42	201.31 ± 7.82***	163.59 ± 6.83***^###^	161.17 ± 10.09***^###&^	143.63 ± 7.20***^###^	
TRACP (ng/mL)	3.75 ± 0.04	4.10 ± 0.13***	3.95 ± 0.07*	4.027 ± 0.05**	3.9 ± 0.11^#^	
BGP (ng/mL)	2.22 ± 0.25	2.27 ± 0.05	2.25 ± 0.09	2.30 ± 0.14	2.26 ± 0.10	
S-CTX (ng/mL)	1.68 ± 0.06	1.88 ± 0.04***	1.83 ± 0.02**	1.80 ± 0.08**	1.74 ± 0.03^##^	

S-Ca: serum calcium; S-P: serum phosphorus; ALP: alkaline phosphatase; TRACP: tartrate-resistant acid phosphatase; BGP: osteocalcin; S-CTX: C-terminal telopeptide of type I collagen

^*^P < 0.05, **P < 0.01, ***P < 0.001 vs. Sham. ^#^*P* < 0.05, ^##^*P* < 0.01, ^###^*P* < 0.001 vs. OVX. ^&^*P* < 0.05 vs RDTF + CaCO_3_

### Effect of RDTF and CaCO_3_ on osteogenesis-related protein expressions

**Fig. 2:**
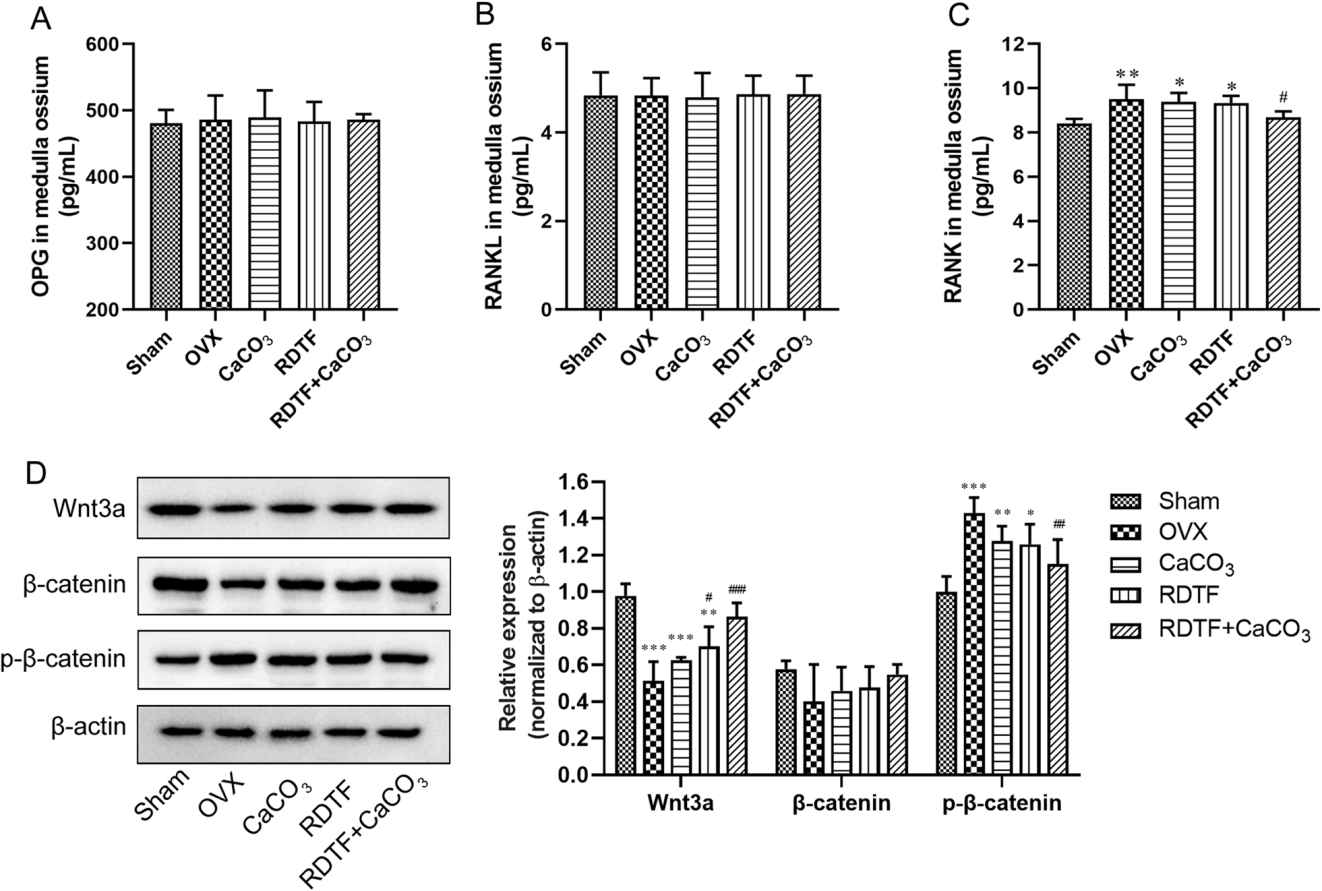
Effect of RDTF and CaCO_3_ on osteogenesis-related protein expressions. **a** Analysis of OPG content in marrow by ELISA. **b** Analysis of RANKL content in marrow by ELISA. **c** Analysis of RANK content in marrow by ELISA. **d** Western blot analysis of Wnt3a, β-catenin, and phosphorylated β-catenin (p-β-catenin) expression in marrow. Error bars indicate SD. *P < 0.05, **P < 0.01, ***P < 0.001 vs. Sham. ^#^*P* < 0.05, ^##^*P* < 0.01, ^###^*P* < 0.001 vs. OVX. ^&^*P* < 0.05, ^&&^*P* < 0.01, ^&&&^*P* < 0.001 vs RDTF + CaCO_3_

The receptor activator of the NF-κB ligand (RANKL) and its decoy receptor osteoprotegerin (OPG) represent osteoblast-derived paracrine cytokines that are essential for osteoclast functions [26, 27]. Monocytic cells differentiate into osteoclasts under the control of macrophage colony-stimulating factor and RANKL, while the monocytic cells differentiation was regulated by OPG via competitively binding to the RANK on the cell surface [28]. There was no significant difference in the level of RANKL and OPG in femoral marrow between Sham group and OVX group, as well as in all the groups (Fig. 2a, b). However, the level of RANK in OVX group was significantly increased compared with Sham group, and only the combined treatment with RDTF and CaCO_3_ could significantly reduce the level of RANK in femoral marrow of OVX rats (Fig. 2c). These data suggested that the anti-osteoporosis effect of the combine treatment with RDTF and CaCO_3_ might be related to the inhibition of RANK expression.

The activation of Wnt3a/β-catenin signal pathway can promote the differentiation of the osteoblast and inhibit the differentiation of osteoclasts by inhibiting the β-catenin phosphorylation-degradation to maintain the transcriptional regulatory activity of β-catenin [29]. As Fig. 2d shows, the OVX treatment significantly down-regulated Wnt3a expression and up-regulated phosphorylated β-catenin (p-β-catenin) expression compared with Sham group, suggesting that the regulating effect of Wnt3a/β-catenin signal pathway on the differentiation of osteoblast and osteoclasts was attenuated, namely OVX-promoted osteoclastic differentiation and the ability of bone resorption by inhibiting Wnt3a/β-catenin signal pathway. However, the activity of Wnt3a/β-catenin signal pathways was improved after these treatments, especially the Wnt3a expression was significantly increased in the RDTF group and the combined treatment with RDTF and CaCO_3_ group, while the p-β-catenin was significantly decreased in the combined treatment with RDTF and CaCO_3_ group. These data indicated that RDTF combination of CaCO_3_ could inhibit osteoclasts differentiation though inhibiting RANK or increasing activity of Wnt3a/β-catenin signal pathway to enhance the anti-osteoporosis effect.

## Discussion

Ovariectomy has shown an increased risk of osteoporosis as occurs with postmenopausal women. In the present study, ovariectomized rats developed bone changes similar to those seen in osteoporotic women as indicated by a decrease in BMD and the fracture of bone trabeculae in femur, a finding that matches with that of Riggs et al., [30], who found that menopause results in elevated bone turnover, an imbalance between bone formation and bone resorption and net bone loss, and this is attributable to the cessation of ovarian function and tapering off estrogen secretion.

Trabecular bone microstructure is considered to be an appropriate predictor of OVX-induced bone loss and bone structural deterioration. We found that RDTF had a positive effect on prevention of OVX-induced fracture of trabecular bone, which was in agreement with the findings of Song et al., [21] who found that bone mass decrease and deterioration of trabecular microarchitecture in ovariectomized rats were improved by high dose of RDTF (≥ 75 mg/kg/d). In the present study, feeding supplemented with CaCO_3_ (20 mg/kg/d) and RDTF (50 mg/kg/d) for 10 weeks exhibited a better and positive effect on prevention of OVX-induced osteoporosis than CaCO_3_ or RDTF alone. CaCO_3_ supplementation increased the parameters of BMD, BMC and BV/TV, but did not improve the fracture of trabecular bone induced by the lack of estrogen. RDTF increased the parameters of BMD, BV/TV, Tb.Th and decreased Tb.Sp parameters, but had no effective effect on BMC parameters. In histopathological examination of femur, the CaCO_3_-treated rats exhibited the same osteoporosis as OVX rats with a sparse and fractured trabecular structure of femur, while RDTF-treated rats exhibited partial trabecular restoration. Notably, RDTF combined with CaCO_3_ showed almost complete recovery of normal structure, with a more effective improvement in the parameters of BMD, BMC, BV/TV, Tb.Th and Tb.Sp than RDTF or CaCO_3_. Therefore, our study indicated that RDTF combined with CaCO_3_ could enhance the anti-osteoporosis effects of RDTF and CaCO_3_ on ovariectomized rats.

Preservation of the trabecular bone architecture significantly promotes bone strength and may be more important in decreasing fracture risk than improving BMD [31]. Although the deterioration of the microstructural geometry of the trabecular bone was largely prevented, the OVX-induced decrease in collagen fibers in femur was not prevented by RDTF, as well as CaCO_3_ supplementation. The inorganic phase of the bone provides the stiffness and the ability to resist compression, whereas the organic phase, mainly constituted of type I collagen, provides bone its flexibility, i.e., the ability to absorb energy and undergo deformation [32, 33]. However, we found that treatment with CaCO_3_ increased the bone mineral content (BMC) in inorganic phase of femur, but could not prevent OVX-induced collagen fibers loss in organic phase, which suggested that calcium supplementations applied to estrogen deficiency-related osteoporosis will increase the risk of fragility fractures for lack of collagen fibers to enhance bone elasticity. Interestingly, Masson’s staining showed no obvious difference in collagen fibers level of femur between OVX rats and CaCO_3_ treated rats, but RDTF and RDTF combined with CaCO_3_ significantly improved the OVX-induced collagen fibers loss. Different treatments caused diverse profiles in bone collagen degradation products, which may have implications for bone quality. How biochemical changes in the bone collagen are associated with fracture and bone quality remains to be investigated and understood.

Studies have found that estrogen plays a vital role in modulating bone remodeling by inducing osteoblast differentiation and reducing osteoclast activity [34, 35]. Attenuated bone formation is an important mechanism in the pathogenesis of postmenopausal osteoporosis, and the loss of ovarian sex steroids at menopause results in accelerated bone turnover with a predominance of bone resorption over bone formation [2, 36]. In our study, OVX accelerated bone turnover and attenuated bone formation with higher ALP and TRACP levels in serum than Sham rats. ALP is regarded as an early marker of osteogenic differentiation [37]. TRACP is an enzyme that is expressed in high amounts by bone-resorbing osteoclasts, inflammatory macrophages, and dendritic cells [38]. However, we did not observe the change of another bone-forming marker BGP after OVX, as well as the treatment of RDTF and CaCO_3_. Thus, OVX promoted the activation of osteoclasts and resulted in accelerated bone turnover with a predominance of bone resorption over bone formation. Moreover, we found that RDTF and CaCO_3_ could inhibit the increase of ALP but not TRACP in serum, while combined treatment with RDTF and CaCO_3_ significantly inhibited OVX-induced TRACP augment. Therefore, our study indicated that RDTF combined with CaCO_3_ could reduce the activity of osteoclasts by inhibiting osteogenic differentiation.

Enhanced bone resorption may be due to accelerated osteoclastogenesis from precursor cells, enhanced fusion and activation of osteoclasts, and prolonged life span due to an inhibition of osteoclast apoptosis [39, 40]. Phytoestrogens is able to enhance osteoblastic OPG production through ER-α-dependent mechanisms and concurrently suppress RANKL gene expression which is associated with an inhibition of osteoclastogenesis [41], and thus estrogen deficiency will induce osteoclastogenesis to aggravate the development of osteoporosis. Furthermore, monocytic cells differentiate into osteoclasts under the control of macrophage colony-stimulating factor and RANKL, while the monocytic cells differentiation was regulated by OPG via competitively binding to the RANK on the cell surface [28]. Song et al. [21] demonstrated that RDTF inhibited osteoclastogenesis via up-regulating OPG and down-regulating RANKL expression. In our study, there was no difference in OPG and RANKL level in femoral marrow between Sham rats and OVX rats, but the RANK level was increased after OVX. Thus, OPG/RANKL unbalance was not the main cause of osteoclastogenesis in our study. Nevertheless, the combine treatment with RDTF and CaCO_3_ significantly reduced the level of RANK in femoral marrow of OVX rats. Therefore, anti-osteoporosis effect of the combine treatment with RDTF and CaCO_3_ might be related to the inhibition of RANK expression.

The activation of Wnt3a/β-catenin signal pathway can promote osteogenic differentiation and inhibit osteoclastogenesis by inhibiting the β-catenin phosphorylation-degradation to maintain the transcriptional regulatory activity of β-catenin [29]. Estrogen deficiency reduces the activation of Wnt3a/β-catenin signal pathway in human osteoblasts (42). Consistent with this result, we found that OVX significantly reduced Wnt3a expression and promoted β-catenin phosphorylation-degradation in the induced osteoblasts. In this study, we found that RDTF attenuated the OVX-induced Wnt3a inhibition and reduced β-catenin phosphorylation-degradation, but the combine treatment with RDTF and CaCO_3_ significantly increased the activation of Wnt3a/β-catenin signal pathway by up-regulating Wnt3a expression and down-regulating phosphorylated β-catenin expression. Therefore, improving the activation of Wnt3a/β-catenin was an important target in anti-osteoporosis effect of RDTF combined with CaCO_3._

## Conclusion

In conclusion, the present data indicated that RDTF combined with CaCO_3_ could effective prevent osteoporosis of ovariectomized rats though acting Wnt3a/β-catenin signal pathway to promote osteogenesis and inhibit osteoclastogenesis. Furthermore, RDTF combined with CaCO3 could prevent OVX-induced decrease in collagen fibers in the femoral tissue of ovariectomized rats and promote the regeneration of new bone or cartilage tissue. Therefore, RDTF combined with CaCO_3_ can be used as a natural approach to help in preventing bone loss associate with states of estrogen deficiency.

## Data Availability

The initial data used to support the findings of this study are available from the corresponding author upon request.

## Abbreviations

OP: Osteoporosis
RDTF: *Rhizoma drynariae* Total flavonoids
CaCO3: Calcium carbonate
OVX: Ovariectomized
OPG: Osteoprotegerin
RANK: Receptor activator of the NF-κB
RANKL: Receptor activator ligand of the NF-κB
p-β-Catenin: Phosphorylated β-catenin
